# Supplementary material for: The complete mitogenome of an unidentified Oikopleura species
Source: F1000Res. 2025 Mar 7;13:1357. Originally published 2024 Nov 12. [Version 2] doi: 10.12688/f1000research.157311.2 (PMC12004063; doi:10.12688/f1000research.157311.2)
Supplement: Peer review history

# Reviewer report: Reviewer response for version 2

**DOI:** 10.5256/f1000research.178729.r374065

**Published:** 2025-04-16

**Authors:** Thomas Stach

**Affiliations:** 1 Humboldt-Universität zu Berlin Vergleichende Elektronenmikroskopie,, Philippstraße, Germany

The manuscript "The complete mitogenome of an unidentified *Oikopleura* species" submitted by J. N. Wibisana and co-authors reports the sequencing of the mitochondrial genome of an unidentified appendicularian species collected off the coast of Japan. The presented mitochondrial genome is new to the respective databases and therefore an original contribution to science. The genome has some features that are evolutionarily interesting and could potentially be used in a cladistic framework. The authors conduct a formal phylogenetic analysis using a Maximum Likelihood algorithm.

 I feel a bit out of my expertise in gauging the validity of the current software used, but based on my experience with other software packages, I would expect that 'ultrafast methods' to calculate bootstrap values overestimate bootstrap support of a node.

 The authors rationalize the study mainly by advancing the knowledge of genome sequences in an underrepresented taxon and the potential use this might have in eDNA research. My main reservation concerning this study is its surprising lack of standard taxonomy approaches. While the sequence as such might be new to science, the species it belongs to, might not be new. Or it might be. From the sequence alone, we cannot know. In order to tie the valuable data generated to the existing body of scientific knowledge, it is - in my view - imperative to at least seriously try to identify the species using the available taxonomic literature on appendicularians. To this end, specimens should have been fixed in a more suitable fixative than Ethanol, in order to preserve the taxonomically relevant characters. They probably should have also been caught more gently as judging from the Figure 1 - but the details of collecting the animals are not given. 

 I have also a few minor comments:

 line 2 in the introduction: it should be 'their life cycle' instead of 'its life cycle'

 third paragraph of the Results and discussion: Oikopleuridae should not be italicized (only Genus and species names are).

 also: '... and misses some of the eight expected ...' I guess the precise number could be stated here. Eight is not so huge that the reader looses track.

 sixth paragraph of the Results and discussion: 'polyphylic' is not the correct term. I assume what is meant is polyphyletic (see also Carmela Gissi's review). But even this is not entirely fitting (at least in the common use of the cladistics terminology), because the depicted phylogeny would be a case of paraphyly, i.e. the genus Oikopleura is paraphyletic in this analysis.

Are the datasets clearly presented in a usable and accessible format, and the assembly and annotation available in an appropriate subject-specific repository?

Yes

Are sufficient details of the sequencing and extraction, software used, and materials provided to allow replication by others?

Partly

Are the rationale for sequencing the genome and the species significance clearly described?

Partly

Are the protocols appropriate and is the work technically sound?

Yes

Reviewer Expertise:

comparative zoology

I confirm that I have read this submission and believe that I have an appropriate level of expertise to confirm that it is of an acceptable scientific standard, however I have significant reservations, as outlined above.

# Reviewer report: Reviewer response for version 2

**DOI:** 10.5256/f1000research.178729.r374064

**Published:** 2025-04-16

**Authors:** Daniel M Chourrout

**Affiliations:** 1 University of Bergen, Bergen, Norway

Although this appendicularian species is not named, having its mt genome sequence  is useful because it contributes to increase taxonomic diversity in phylogenetic trees. The annotation is carefully performed and the conclusions make sense, with a clear position next to O. longicauda and two giant larvaceans. I have two comments:

 1. Why not comparing the order of the coding genes with other appendicularian species ? Previous work on other species shows that synteny is not conserved with other tunicates and it could be the main contribution of the paper if this comparison was performed.

 2. The picture is of extremely poor quality and it should not be index together with the main text. It may be sent to an annex as it does not bring information except that the species is an appendicularian, this is clear enough from the phylogenetic analysis. How a better picture could not be obtained is surprising, it might have helped to tentatively name the species.

Are the datasets clearly presented in a usable and accessible format, and the assembly and annotation available in an appropriate subject-specific repository?

Yes

Are sufficient details of the sequencing and extraction, software used, and materials provided to allow replication by others?

Yes

Are the rationale for sequencing the genome and the species significance clearly described?

Yes

Are the protocols appropriate and is the work technically sound?

Yes

Reviewer Expertise:

animal genomics

I confirm that I have read this submission and believe that I have an appropriate level of expertise to confirm that it is of an acceptable scientific standard.

# Reviewer report: Reviewer response for version 1

**DOI:** 10.5256/f1000research.172738.r341076

**Published:** 2025-01-06

**Authors:** Ayelet Voskoboynik

**Affiliations:** 1 Stanford University, Stanford, USA

This paper presents the mitochondrial genome assembly and annotation of an unknown appendicularian species collected from Japan. The findings contribute valuable data for future eDNA studies.

 The paper includes a thorough analysis of the mitochondrial genome including annotation and phylogenetics analyses.

 However, the paper is missing important metrics regarding the PacBio Sequel II sequencing results. Please address the following points  to make the article scientifically sound: 

 1. Add the following metrics regarding the PacBio Sequel II sequencing results: HiFi Reads, HiFi Yield (bp), HiFi Reads Length (Mean bp), HiFi Read Quality (median), HiFi Number of Passes (mean), HiFi Reads Length Summary, HiFi Read Length Distribution.

 2. Also include the stat for the assembly itself including: Contig type (Primary vs haplotigs; polished contigs; max contigs length; median contig length, N50 Contig Length, Sum of Contig length.

 3. PacBio assemblers obtain circular contigs which include the mitochondria DNA (and bacteria DNA). If circular contigs were not utilized to directly obtain mtDNA, a brief explanation of the rationale for this decision would be valuable.

Are the datasets clearly presented in a usable and accessible format, and the assembly and annotation available in an appropriate subject-specific repository?

Yes

Are sufficient details of the sequencing and extraction, software used, and materials provided to allow replication by others?

Partly

Are the rationale for sequencing the genome and the species significance clearly described?

Yes

Are the protocols appropriate and is the work technically sound?

Partly

Reviewer Expertise:

genomic, evolution, comparative immunology and stem cell biology

I confirm that I have read this submission and believe that I have an appropriate level of expertise to confirm that it is of an acceptable scientific standard.

# Response

**Published:** 2025-02-27

**Authors:** Johannes Nicolaus Wibisana

*This paper presents the mitochondrial genome assembly and annotation of an unknown appendicularian species collected from Japan. The findings contribute valuable data for future eDNA studies.*

*The paper includes a thorough analysis of the mitochondrial genome including annotation and phylogenetics analyses.*

*However, the paper is missing important metrics regarding the PacBio Sequel II sequencing results. Please address the following points  to make the article scientifically sound:*

*1. Add the following metrics regarding the PacBio Sequel II sequencing results: HiFi Reads, HiFi Yield (bp), HiFi Reads Length (Mean bp), HiFi Read Quality (median), HiFi Number of Passes (mean), HiFi Reads Length Summary, HiFi Read Length Distribution.*

*2. Also include the stat for the assembly itself including: Contig type (Primary vs haplotigs; polished contigs; max contigs length; median contig length, N50 Contig Length, Sum of Contig length.*

*3. PacBio assemblers obtain circular contigs which include the mitochondria DNA (and bacteria DNA). If circular contigs were not utilized to directly obtain mtDNA, a brief explanation of the rationale for this decision would be valuable.*

 Response:

 Thank you for the comments. We have added the PacBio sequencing metrics to the supplementary files.

 We have also added to the method section the method for the fragmentation step, which enables mtDNA sequencing on the PacBio platform. The mitochondrial genome presented here was produced with the NOVOLoci software in targeted assembly mode, which yielded a linear concatenate sequence which we circularised by hand.

 These changes have been reflected in the manuscript as follows:

 “The sequenced reads were assembled with NOVOLoci ( https://github.com/ndierckx/NOVOLoci) in targeted assembly mode.”

 “DNA was fragmented using Megaruptor 3® (Diagenode, USA) using the Megaruptor 3 Shearing Kit (Diagenode, USA #E07010003) at speed 32 and purified using SMRTbell cleanup beads (Pacific Biosciences, USA #102-158-300). Quality control of obtained DNA was performed using the Femto Pulse System (Agilent, USA) and the Genomic DNA 165 kb Kit (Agilent, USA #FP-1002-0275). The sequencing was performed on a PacBio ® Sequel II sequencer (Pacific Biosciences, USA) using the Sequel II sequencing kit 2.0 (Pacific Biosciences, USA #101-820-200). The DNA size profile and sequencing metrics are available on Zenodo (doi: 10.5281/zenodo.14934254).”

# Reviewer report: Reviewer response for version 1

**DOI:** 10.5256/f1000research.172738.r341080

**Published:** 2025-01-06

**Authors:** Carmela Gissi

**Affiliations:** 1 Department of Biosciences, Biotechnology and Environment, Università degli Studi di Bari Aldo Moro, Bari, Italy

The paper describes the sequencing and assembly of the mitochondrial genome of a larvacean of the genus Oikopleura and also reports the mitochondrial phylogenetic analysis of Larvacea together with other chordate representatives. The manuscript is clear and well written, but need to be improved with details on the sample and the applied protocols as well as with additional evolutionary analyses

 I suggest to better stress in the introduction the need to sequence other larvacean representatives, not only for eDNA studies but also for evolutionary studies aiming at understanding the origin of chordates and tunicates, and to unravel the hidden biodiversity of Larvacea, a quite neglected taxon

 Was the DNA extracted from a single specimen preserved in 99% ethanol or at -80 °C?

 In the Methods section, the Authors reports that "Quality control of obtained DNA was performed using Agilent 4200 TapeStation (Agilent, USA). The sequencing was performed on a PacBio ^®^ Sequel II sequencer (Pacific Biosciences, USA)." Further details should be added. In particular, the Authors should report the ng and quality (i.e., absorbance values, TapeStation fragment profile) of the extracted DNA, the used PacBio library and sequencing kits, and the PacBio protocol used. Indeed, I suppose it was used the PacBio ultra-low input protocol, and, if so, how was the DNA fragmented?

 As for the phylogenetic tree, it seems that it was reconstructed using all 13 protein-coding genes, including those absent in larvaceans. Moreover, the tree was reconstructed from nucleotide, not from amino acid sequences, as is common when long evolutionary distances are analyzed, in order to avoid substitution saturation phenomena. I suggest reconstructing the tree also from amino acid sequences, with/without the protein-genes absent in larvaceans, so considering only positions without gaps and then to compare the obtained trees. Bayesian trees should also be reconstructed to confirm the obtained phylogeny

 A well formatted "full version" of the tree of Figure 3 should be provided in the Supplementary Material. Although out of the scope of the paper, a brief comment on the recovered tunicate phylogeny should be reported

 The Zenodo Supplementary material contains a folder with the results of IQ-TREE and RaxML analyses: could you please comment if there were differences between the trees obtained with the two methods?

 For not-users of the IQ-TREE software, I suggest explaining the meaning of the used command-line options and reporting that this software performs ML tree reconstructions

 The Authors should clarify the legend of Figure 3, including the number of genes analysed, the length of the alignment, the method and model used for the tree reconstruction.

 "Possible explanations for this result are heteroplasmy or that the mitogenome consists of concatemers ": please explain better why you hypothesize the existence of concatemers.

 "As the Oikopleura genus is polyphylic in our phylogenetic analysis and that of others,..." : the Authors should cite the references where the Oikopleura genus was already found as polyphyletic

 "Furthermore, these two haplotypes are supported by homopolymer variants at multiple different loci": a table with all variants between the two haplotypes should be reported in the Supplementary Material, particularly if Figure 2 shows only a portion of these variants.

 I suggest making available in NCBI also the second assembled haplotype

Are the datasets clearly presented in a usable and accessible format, and the assembly and annotation available in an appropriate subject-specific repository?

Yes

Are sufficient details of the sequencing and extraction, software used, and materials provided to allow replication by others?

Partly

Are the rationale for sequencing the genome and the species significance clearly described?

Partly

Are the protocols appropriate and is the work technically sound?

Partly

Reviewer Expertise:

mitogenomics, tunicate evolution, long read NGS

I confirm that I have read this submission and believe that I have an appropriate level of expertise to confirm that it is of an acceptable scientific standard, however I have significant reservations, as outlined above.

# Response

**Published:** 2025-02-27

**Authors:** Johannes Nicolaus Wibisana

*I suggest to better stress in the introduction the need to sequence other larvacean representatives, not only for eDNA studies but also for evolutionary studies aiming at understanding the origin of chordates and tunicates, and to unravel the hidden biodiversity of Larvacea, a quite neglected taxon*

 Response:

 Thank you for this comment. We have added the following statement in the introduction:

 “In addition to that, mitochondrial DNA sequence of larvaceans can be useful in evolutionary studies to the origin of tunicates, as well as chordates, and as a means to understand the hidden biodiversity of Larvacea, which is a quite neglected taxon.”

*Was the DNA extracted from a single specimen preserved in 99% ethanol or at -80 °C?*

 Response:

 Thank you for this comment. We preserved the specimen in 99% ethanol at -80°C. We have added the following statement to the manuscript:

 “Samples were preserved in 99% ethanol at -80°C prior to DNA extraction.”

*In the Methods section, the Authors reports that "Quality control of obtained DNA was performed using Agilent 4200 TapeStation (Agilent, USA). The sequencing was performed on a PacBio® Sequel II sequencer (Pacific Biosciences, USA)." Further details should be added. In particular, the Authors should report the ng and quality (i.e., absorbance values, TapeStation fragment profile) of the extracted DNA, the used PacBio library and sequencing kits, and the PacBio protocol used. Indeed, I suppose it was used the PacBio ultra-low input protocol, and, if so, how was the DNA fragmented?*

 Response:

 Thank you for pointing this out. We have revised the quality control method, which used the Femto Pulse system after fragmentation. We also added the fragmentation protocol to the method section. As for the profile of the DNA fragments, we have attached the Femto Pulse run report to the supplementary files on Zenodo. We have adjusted the text as follows:

 “DNA was fragmented using Megaruptor 3 ® (Diagenode, USA) using the Megaruptor 3 Shearing Kit (Diagenode, USA #E07010003) at speed 32 and purified using SMRTbell cleanup beads (Pacific Biosciences, USA #102-158-300). Quality control of obtained DNA was performed using the Femto Pulse System (Agilent, USA) and the Genomic DNA 165 kb Kit (Agilent, USA #FP-1002-0275). The sequencing was performed on a PacBio ® Sequel II sequencer (Pacific Biosciences, USA) using the Sequel II sequencing kit 2.0 (Pacific Biosciences, USA #101-820-200). The DNA size profile and sequencing metrics are available on Zenodo (doi: 10.5281/zenodo.14934254).”

*As for the phylogenetic tree, it seems that it was reconstructed using all 13 protein-coding genes, including those absent in larvaceans. Moreover, the tree was reconstructed from nucleotide, not from amino acid sequences, as is common when long evolutionary distances are analyzed, in order to avoid substitution saturation phenomena. I suggest reconstructing the tree also from amino acid sequences, with/without the protein-genes absent in larvaceans, so considering only positions without gaps and then to compare the obtained trees. Bayesian trees should also be reconstructed to confirm the obtained phylogeny*

 We used nucleotides instead of amino acids because we are still uncertain on how to translate the TGA codon in the *Mesochordaeus *and *Bathochordaeus *mitogenomes (Pichon et al., 2020), and worried that it may affect the results. Following the reviewer’s suggestion, we computed trees from amino acid sequences using the same IQ-TREE command line as for nucleotide sequences (as it selects the best models automatically), with or without the proteins that are not found in the appendicularian mitogenomes. In both cases the results were consistent with the nucleotide tree, by placing the new mitogenome in the same clade as *O. longicauda*, placing the appendicularians as a sister clade to thaliaceans except for *D. nationalis*, and identifying *Ciona *as a clade distinct from the other phlebobranchians. Unfortunately, the amino acid-based trees did not recover the expected relation between the three chordate sub-phyla nor between tunicate classes. We added a copy of these computations to our supplemental material for the reviewers and readers convenience, nevertheless we do not provide guarantee that the trees can be useful for other purposes than accountability of our work for this revision. We also did not compute Bayesian trees for this revision due to the lack of expertise in our team.

*A well formatted "full version" of the tree of Figure 3 should be provided in the Supplementary Material*

 We have added the full version of the tree in the supplementary material under Figure S4.

*Although out of the scope of the paper, a brief comment on the recovered tunicate phylogeny should be reported*

 We added the following: “Considering the tunicates phylogeny, the tree recovered clades for the free-living Appendicularia, Thaliacea, and for the sessile Stolidobranchia, Aplousobranchia and Phlebobranchia, from which it detached the *Ciona *genus as a separate clade. A single thaliacean species, *Doliolum nationalis*, grouped with the sessile tunicates, however this is not fully supported by bootstrap values. This computed tree suggests that targeted sampling and sequencing of additional doliolids and *Ciona *species may be useful to further clarify the phylogeny of tunicates classes and orders.”

*The Zenodo Supplementary material contains a folder with the results of IQ-TREE and RaxML analyses: could you please comment if there were differences between the trees obtained with the two methods?*

 The two trees have very similar topologies (identical when folded like in Figure 3), and we chose the IQ-TREE one because it provides bootstrap values that are easy to interpret.

*For not-users of the IQ-TREE software, I suggest explaining the meaning of the used command-line options and reporting that this software performs ML tree reconstructions*

 We changed the method section’s text to “-T AUTO (run as many CPU threads as possible) --runs 3 (perform 3 runs to assess model convergence) --polytomy (allow unresolved branches) --ufboot 1000 (1000 boostraps with the ultrafast method) -m MFP (automatic selection of the best model for maximum likelihood inference using the ModelFinder Plus method”

*The Authors should clarify the legend of Figure 3, including the number of genes analysed, the length of the alignment, the method and model used for the tree reconstruction.*

 We changed the figure legend to “Phylogenetic tree computed by maximum likelihood inference on a ~13 kbp codon alignment of 13 mitochondrial genome protein-coding genes collected from publicly available aquatic chordate genomes”. We did not indicate the model as we used one partition per gene and the software did not chose the same model for each partition.

*"Possible explanations for this result are heteroplasmy or that the mitogenome consists of concatemers ": please explain better why you hypothesize the existence of concatemers.*

*"Furthermore, these two haplotypes are supported by homopolymer variants at multiple different loci": a table with all variants between the two haplotypes should be reported in the Supplementary Material, particularly if Figure 2 shows only a portion of these variants.*

*I suggest making available in NCBI also the second assembled haplotype*

 Response:

 Thank you for this comment. We have reanalyzed the sequences and we could not verify if the observed variation were true variants or caused by sequencing errors in homopolymers and therefore decided to remove the hypothesis of a second haplotype. The one haplotype is the one that is published online. We apologize for this error, and we confirmed that the current available sequence on NCBI is the correct one. We have also modified the text to remove the parts referencing the haplotypes.

*"As the Oikopleura genus is polyphylic in our phylogenetic analysis and that of others,..." : the Authors should cite the references where the Oikopleura genus was already found as polyphyletic*

 Response:

 Thank you for this comment. We have added the following references to support this statement:

- Galt CP, Grober MS, Sykes PF. Taxonomic Correlates of Bioluminescence Among Appendicularians (Urochordata: Larvacea). The Biological Bulletin. 1985 Feb;168(1):125–34.
- Naville M, Henriet S, Warren I, Sumic S, Reeve M, Volff JN, et al. Massive Changes of Genome Size Driven by Expansions of Non-autonomous Transposable Elements. Current Biology. 2019 Apr;29(7):1161-1168.e6.
- Masunaga A, Mansfield MJ, Tan Y, Liu AW, Bliznina A, Barzaghi P, et al. The cosmopolitan appendicularian Oikopleura dioica reveals hidden genetic diversity around the globe. Mar Biol. 2022 Nov 27;169(12):157.
- Plessy C, Mansfield MJ, Bliznina A, Masunaga A, West C, Tan Y, et al. Extreme genome scrambling in marine planktonic Oikopleura dioica cryptic species. Genome Res. 2024 Apr 25;34(3):426–40
